# Neuroscientists’ Classroom Visits Positively Impact Student Attitudes

**DOI:** 10.1371/journal.pone.0084035

**Published:** 2013-12-16

**Authors:** Janet L. Fitzakerley, Michael L. Michlin, John Paton, Janet M. Dubinsky

**Affiliations:** 1 Biomedical Sciences Department, University of Minnesota, Duluth, Minnesota, United States of America; 2 Department of Neuroscience, University of Minnesota, Minneapolis, Minnesota, United States of America; 3 Center for Applied Research and Educational Improvement, University of Minnesota, St. Paul, Minnesota, United States of America; The University of Western Ontario, Canada

## Abstract

The primary recommendation of the 2010 President’s Council of Advisors on Science and Technology report on K-12 education was to inspire more students so that they are motivated to study science. Scientists’ visits to classrooms are intended to inspire learners and increase their interest in science, but verifications of this impact are largely qualitative. Our primary goal was to evaluate the impact of a longstanding Brain Awareness classroom visit program focused on increasing learners understanding of their own brains. Educational psychologists have established that neuroscience training sessions can improve academic performance and shift attitudes of students from a fixed mindset to a growth mindset. Our secondary goal was to determine whether short interactive Brain Awareness scientist-in-the-classroom sessions could similarly alter learners’ perceptions of their own potential to learn. Teacher and student surveys were administered in 4^th^-6^th^ grade classrooms throughout Minnesota either before or after one-hour Brain Awareness sessions that engaged students in activities related to brain function. Teachers rated the Brain Awareness program as very valuable and said that the visits stimulated students’ interest in the brain and in science. Student surveys probed general attitudes towards science and their knowledge of neuroscience concepts (particularly the ability of the brain to change). Significant favorable improvements were found on 10 of 18 survey statements. Factor analyses of 4805 responses demonstrated that Brain Awareness presentations increased positive attitudes toward science and improved agreement with statements related to growth mindset. Overall effect sizes were small, consistent with the short length of the presentations. Thus, the impact of Brain Awareness presentations was positive and proportional to the efforts expended, demonstrating that short, scientist-in-the-classroom visits can make a positive contribution to primary school students’ attitudes toward science and learning.

## Introduction

### Scientists-in-the-classroom (SiC)

 As part of the responsible conduct of research, scientists are expected to communicate their findings to the lay public and promote public understanding of the science and its policy implications [1]. Moreover, scientists are expected to exert leadership by stimulating interest in science and developing scientific expertise among K-12 science teachers [2]. One entry point for the prescribed US effort to increase teacher professional development in science is for scientists to visit classrooms [2]. While this was never intended to be the only point of contact, classroom visits are considered essential for introducing scientists to the challenges of actively teaching science at the pre-collegiate level and for supporting teachers who had received more in-depth professional development [2]. Single classroom visits by scientists often do not adequately address specific content or concepts that are aligned with state or national standards, but rather focus on individual scientist’s personal expertise. Some programs carefully align curricula to students needs and educational standards [3], while others promote science related to the originating organizations’ core mission or strengths [4–6]. While classroom visits are considered a starting place, developing teacher expertise in scientific practices and specific content areas is viewed as a more intense, long term development process.

 Although it is important that the scientific community engages more fully in pre-service and in-service science teacher education, SiC programs have an important role. The initial, primary recommendation of the 2010 President’s Council of Advisors on Science and Technology report on K-12 Education [7] was *to inspire* more students so that they are motivated to study more science. When interviewed in a qualitative evaluation of one such SiC program, teachers indicated that one of the primary benefits was enhanced interest and engagement across all student ability levels [8]. Teachers felt scientist visits dispelled stereotypes and provided students with an increased understanding of scientific concepts, skills, and relevance [8]. Thus, even short classroom visits were felt to be beneficial and may align with the stated national goal to inspire the next generation of scientists. 

 Hard data, however, on the impact that such visits have on students is largely absent from the evaluation and educational literature. In a recent exploratory study of the public engagement activities of European research institutions, no monitoring or evaluation measures were in place for any of the 40 institutions, the majority of which had organized initiatives aimed at schools [9]. Following the initial survey, interviews were conducted at 12 institutions. Of these, only one had active evaluation measures in place. The other 11 institutions acknowledged that evaluations might be useful in guiding programmatic choices [9]. Among scientists from top US research institutions surveyed in 2009-2010, 58% were engaged in some sort of public outreach activity. The majority of these (32%) were involved in K-12 classroom visits [10]. Given that classroom interactions remain a common forum for translation of scientific information to the public, assessment of SiC programs becomes necessary to determine if these efforts are effective.

### Brain Awareness

 The Brain Awareness (BA) campaign (previously called Brain Awareness Week) was initiated by the Dana Alliance for Brain Initiatives and the Society for Neuroscience (SfN) in 1996 to promote awareness of issues related to brain health and disease and the benefits of neuroscience research [5,11]. Public understanding of brain function is fragmented and has not kept up with the rapid expansion of scientific knowledge regarding detailed molecular, cell biological and systems functioning within the nervous system [12–15]. Exaggerated and incomplete communication of research findings has resulted in an abundance of brain myths [16–18]. Similarly, coupling a brain image with other inaccurate information makes the latter more credible [19]. Established scientific knowledge regarding such important concepts as learning and memory have not been adequately communicated to the public, nor are they adequately taught in schools [15,20,21]. 

 Brain Awareness was conceived as a public health campaign combined with outreach by the neuroscientific community to inform the public regarding the growing predominance of neurodegenerative and developmental disorders. Neuroscientists were encouraged by SfN to develop local activities such as public lectures, brain fairs and school visits to deliver messages conveying the excitement for research in the field of neuroscience with basic understandings of how the brain works. Scientists worldwide have organized a variety of activities aimed at both the general public and school audiences [22]. These endeavors have generated energy and commitment within the neuroscientific community to translate our findings to the public as evidenced by the ongoing nature of these yearly events [5,11]. Local programs vary including large scale public lectures [23], open houses [24], brain fairs [25], museum events and classroom visits [26]. However, like many well intentioned efforts by scientists to engage the public, BA activities and events have largely been judged on reports from the presenters themselves and not on any unbiased assessment of how audiences responded or what audiences understood (e.g. links within [22,23]). The neuroscience community at the University of Minnesota (UMN) has been involved with the BA campaign from its inception in 1996. Beginning in 1998, our program initiated SiC visits as a means to involve and train graduate students in public engagement. In 2005, the program was expanded to medical students. Mindful of the general lack of evaluation of SiC programs and the specific need for evaluation of BA, we designed and conducted an outcome evaluation of our BA SiC program in the state of MN.

### Theories of Intelligence

 Understanding that the brain physically changes during learning and that one can effect such changes with effort has become the foundation for many theories of change and their application [27]. Understanding how learning and memory are constructed from one’s own repeated and relevant experiences adds to students’ metacognitive knowledge, an essential component for educational success [28]. Communicating to elementary students that their brains are plastic and their effort matters may build their resilience for later educational encounters with courses that emphasize content knowledge acquisition [29]. Introducing the concepts that 1) brains change with learning and 2) this requires effort on the part of the learner shifts students from embracing an entity theory of intelligence (a belief that their intelligence is fixed), to an incremental theory of intelligence (a belief that intelligence is malleable) [30]. Indeed, developing individual students’ self-identity as learners, in general, and as scientists, specifically, may contribute to development of their own science identities [31]. 

### Purpose of study

 In designing this outcome evaluation, we sought measures that would reflect factors that could be impacted by the short nature of a SiC visit. At the most basic level, we needed to assess if students understood and remembered the neuroscience content we presented. A previous report that a short 20 min lesson on sensory perception and the brain for first graders produced significant retention of understanding of a range of brain functions three weeks later [21], provided an example of what we might accomplish. Additionally, we choose to assess attitudes towards science since this was one of the primary foci of the national calls for scientists to engage the public [1,2]. Lastly, as part of the content assessment, we determined whether our classroom visits could alter student attitudes about their own ability to learn [30].

 Student attitudes toward science and interest in science have been tracked historically in many ways and appear to be influenced by attitudes of teachers, parents, peers, culture and the media and the quality and manner in which science is taught in schools [32]. Student attitudes towards science are influenced by their own self-concept of how capable they are in science (self-efficacy), their view of the usefulness of science, peer attitudes, teacher enthusiasm, and *implementation* and encouragement of science activities rather than memorization or *participation* in science activities [32,33]. Expressing interest in a scientific career at grade 8 predicted eventual successful completion of a science or engineering baccalaureate degree [34]. Attitudes towards science are multidimensional, containing affective, cognitive and behavioral components that may be directed towards specific objects, for example interest in science, motivation towards science, enjoyment of science, perceptions of scientists, self-esteem for science or attitudes towards a specific area of science [32,35]. Student attitudes toward science decline over middle school years [32,33]. Involving students in two week summer science experiences slowed the longitudinal decline in attitude towards science across the middle to high school transition [36]. In the context of scientist classroom visits, if the presenter employs unique, age-appropriate, stimulating activities to engage students in doing science, the deviation from classroom routine becomes welcome and may spark interest [8,37,38]. 

 Since the current US National Science Education Standards (NSES) do not emphasize neuroscience, students can traverse the educational system without a strong conceptual understanding of how learning occurs in the brain [15]. The UMN Brain Awareness classroom visits introduce these concepts to upper elementary students. In Blackwell et al.’s (2007) study establishing that teaching students how the brain learns changes mindsets and improves performance on standardized tests, the neuroscience intervention occurred once a week for eight weeks [30]. The Brain Awareness visits are a one hour presentation, thus we did not know if the weaker ‘strength’ of our SiC intervention would have a measureable impact. Could the short presentation of neuroscience content result in changing student mindsets as defined above? As this idea was part of the content delivered, it merited inclusion in the evaluation of how memorable the SiC visits were. Since the content message focused upon the biological basis of learning, we explored whether the presentations could alter students attitudes towards their own abilities to learn.

 Thus, student general attitude towards science [32,33] and student mindset (fixed vs. growth)[30] emerged as two domains that might be influenced by presenting brains and active examples of nervous system function in classrooms. Based on that perspective, we developed three foundational but overlapping research questions that were evaluated in our survey:

1. Do the BA classroom visits present neuroscience concepts (particularly ones related to synaptic plasticity) that are valued by teachers and remembered by upper elementary audiences?
2. Does a short encounter with the idea that synapses change with learning enhance students’ concepts of their own potential to grow intellectually (growth mindset) and diminish their identification with a limited intellectual growth potential (fixed mindset)?
3. Do neuroscientists’ classroom visits alter students’ attitudes towards and general interest in science?

Positive responses to these questions emerged from a local study of the Minnesota BA program conducted in the Twin Cities in 2010 and a larger state-wide evaluation performed in 2011.

## Methods

### Program Description

The overall goals of the University of Minnesota Brain Awareness program are to increase students’ appreciation of their own ability to learn and to contribute to their general understanding of basic brain function. To accomplish this, scientists and students from the Twin Cities (TC) and Duluth campuses of the University of Minnesota visited grade 4 through 6 classrooms with 45-60 minute interactive presentations. In Duluth and TC, solicitations were sent broadly to all schools within a restricted traveling distance and visits were scheduled with those that responded. In greater MN, presenters contacted schools and made individual scheduling arrangements. A typical presentation included a short introduction about the presenter and the University, interactive demonstrations, and real human and animal brains. The broad neuroscience concepts that were covered included structure-function relationships for large brain areas, the idea of electrical and chemical communication and the concept that learning changes the connections in the brain. The concept of a neuron and its specialized structure was introduced only if students understood cells as a basic unit of living things. Synaptic plasticity as the basis for learning and memory is an area of neuroscience not well appreciated by the public at large [13]. Besides being relevant to education, learning and memory is one of the five most frequently requested topics in a survey of visitors to a Brazilian neuroscience website [12]. Many demonstrations were designed to teach concepts of synaptic plasticity, by showing how the brain can change in an activity-dependent way [39,40]. A large repertoire of different options was available to all presenters, who chose which specific activities to do in their particular classroom. Data in this study are based on 168 presentations in 2011; 107 were made in the Minneapolis/St. Paul metropolitan area (Twin Cities, TC) and in Duluth (combined as “urban”) and 61 throughout greater Minnesota (“rural”). In 2010, surveys were distributed to 52 classrooms (pre-survey), 54 classrooms (post-survey) in 21 schools visited by 45 presenters in the Twin Cities.

### Presenter Training procedures

 Presenters were from all academic ranks: faculty, lab staff and graduate, medical and undergraduate students. Scientific presenters in the BA program were not expected to have prior experience or skills in interacting with K-12 students or teachers as most scientists do not receive instruction in educational issues during their training [4,41]. All presenters received a minimum of one hour of explanatory training and spent additional time planning the activities they wanted to use [41]. Training of presenters in Duluth emphasized doing at least one of three main activities which would illustrate the idea of synaptic plasticity. Training of presenters in the TC focused upon introducing all the activities available, how to illustrate the main points, how to control a classroom and how to tailor the presentation to respond to student questions. Duluth presenters and the Duluth medical students were required to be trained prior to going into the schools for the years in this survey. In the TC, only new presenters were required to attend the training sessions. Anyone who had gone out in previous years was considered to be “experienced.” Presenters from the scientific communities in the TC went into classrooms in pairs, or in some cases as a group from the same lab. First time presenters were paired with an experienced presenter. Presenters visiting schools in greater MN and Duluth usually went into classrooms individually.

### Surveys

 To assess the impact of the classroom visits, both teachers and students were surveyed in the 2010-11 and 2011-12 academic years. Teachers were asked to rate the value of BA presentations in two ways: first, by indicating their degree of agreement with six statements on three-point Likert-type scales (1 = “not valuable”; 2 = “somewhat valuable”; 3 = “very valuable”); second, by answering two open-ended questions regarding the positive and negative aspects of the BA presentations. Teachers were also asked to provide information regarding their teaching experience. Post-visit teacher surveys were returned from 147 4-6 grade teachers from 2010 and 2011. 

 We conducted an initial student survey in 2010 that was Twin Cities centric. All qualitative responses are based on that 2010 dataset. All quantitative results (particularly the factor analysis) are based on the 2011 dataset. In 2010, 2655 primary school surveys that were administered to Twin Cities students were evaluated: 1111 (41.8%) pre-presentation surveys and 1544 (58.2%) post-presentation surveys. In 2011, 4,805 primary school surveys were evaluated: 2150 (45%) pre-presentation surveys and 2,655 (55%) post-presentation surveys. 

 The eighteen forced-choice items for the student survey were adapted from or modeled after previous student science interest surveys [32,33,36,42] and were evaluated by neuroscientists and education specialists before 2010 in order to chose items that represented ideas regarding upper elementary students’ attitudes towards science, views of scientists, and their own ability to learn. Additional evaluation of the face validity of the factor analysis was performed by a different group in 2013 (see results). Statements for this last concept were adapted from the work of Carol Dweck [43]. The forced-choice items were expected to address research questions two and three. Students indicated their degree of agreement with the statements on five-point Likert-type scales ranging from 1 = “strongly disagree” to 5 = “strongly agree.” Approximately half of the classrooms were randomly assigned to be surveyed prior to the visit, while students in the remaining classrooms filled out the surveys after the visit. Students filled out either the pre- or post-presentation survey, but not both. The timing of the post-visit survey was determined by the host teacher, typically within a few days of the visit. Classroom scheduling always involves last minute changes, therefore, the pre- and post-visit survey distribution was not balanced with respect to school demographics. 4,805 primary school surveys were evaluated: 2150 (45%) pre-presentation surveys and 2,655 (55%) post-presentation surveys. In addition, primary school students were asked two open-ended questions regarding the Brain Awareness visits. No attempt was made to balance the survey distribution based upon demographics or the experience of the presenters. 

### Ethics Statement

 The University of Minnesota (UMN) IRB advised that this study was exempt since this was an evaluation of an educational experience, survey responses were totally anonymous, no personal information was being collected on either students or teachers, and only aggregate data were being reported. In 2011, school name was associated with each survey. 

### Demographics and Statistics

 Schools participating in the 2011 survey data were categorized using publicly available demographic data from the National Center for Education Statistics (NCES, http://nces.ed.gov/). Independent schools do not report free or reduced lunch data, but many voluntarily report racial/ethnic breakdowns. Therefore, diversity, but not poverty, information was available for many independent school classrooms. A school’s poverty rating was based on the percentage of students eligible for free or reduced price lunch: high poverty > 75%, medium = 25-75%, low < 25%. A school’s diversity rating was based on the percentage of students in the building that identified in the nonwhite racial/ethnic categories: high diversity > 75%, medium = 25-75%, low < 25%. There was not a balanced representation of school demographics in each category.

 All statistical calculations were made with IBM SPSS 19 statistics software. The effect sizes were calculated either on a Web site calculator (http://www.uccs.edu/~faculty/lbecker/) or, more frequently, in Excel. 

 Prior to performing the factor analysis, the Kaiser-Meyer-Olkin (KMO) Measure of Sampling Adequacy yielded a value of 0.84, justifying this approach. In addition, Bartlett's Test of Sphericity (Approx. Chi-Square = 4907.7, *df* = 153, *p* < .001) confirmed a strong relationship among the survey items and indicated the data were not uncorrelated. Principal axis factoring with direct oblimin rotation on the post-presentation data was used to generate a reduced set of variables in the exploratory factor analyses. An oblique rotation (direct oblimin) was performed on all factors with eigenvalues of 1.0 or larger. An orthogonal solution (e.g., typical Varimax rotation) of the resultant factor structure was not forced as the conceptual categories of the 18 survey items did not suggest orthogonality. The resultant rotated pattern matrix yielded a relatively clean set of factor loadings. For the resultant factors, the composite variables were computed by summing the items that loaded onto each factor and then dividing by the number of items in the factor, to keep the scores in the same range (1 to 5) as the original items. 

## Results

### Teacher views of outcomes

 Two-thirds of the teachers responding to the survey had masters degrees, with the remainder having bachelors degrees. The teachers who participated in Brain Awareness averaged 18.6 ± 9.6 years of teaching experience following initial training. There was no significant difference in years in service between teachers in urban and rural Minnesota (Mann-Whitney Rank sum test; p=0.693). Ninety-five percent of the teachers did not have any specialized experience or training in neuroscience. One teacher had attended the BrainU teacher professional development program through UMN [44]. 

**Figure 1:**
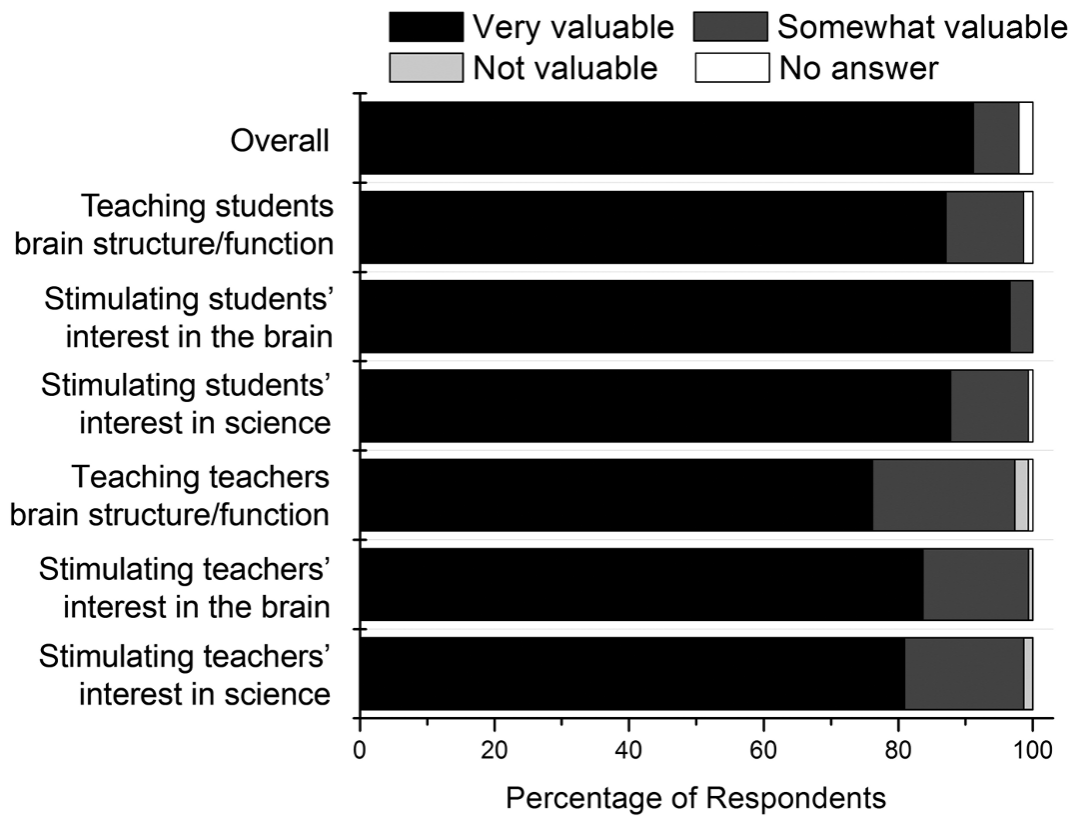
Teacher responses to forced choice questions regarding the value of the BA presentations. Data represent the percent of teacher responses in each category of the 3 point scale: 1= not valuable, 2 = somewhat valuable, 3 = very valuable.

 Teachers found the Brain Awareness presentations to be a valuable experience (Figure 1), with 90% of teachers rating the program as very valuable in overall impact and >95% saying that the visits stimulated students’ interest in the brain. The BA visits also had a beneficial effect on the teachers themselves, with 75-85% of the teachers reporting that the presentations stimulated their interest in the brain and in science, and taught them new things about brain structure and function.

 When asked open-ended questions regarding positive and negative aspects of the Brain Awareness visits (Table 1), 98% of the teachers provided 290 positive comments (~2/teacher). The most common responses related to the benefits of hands-on, age-appropriate activities and of bringing actual brain specimens into the classroom. Teachers indicated that these activities resulted in enthusiastic participation of the primary school students in the BA presentations. Eighty-one percent either explicitly stated that there were no negative aspects related to the presentations (86/147) or did not provide any negative comments (33/147). Negative comments (26 total, ~0.2/teacher) focused upon presenter or organization related issues (e.g. activities got out of hand). Overall, the teachers’ qualitative comments supported their assessment of the Brain Awareness presentations as valuable.

**Table 1:** Teacher comments about Brain Awareness presentations.

**Question**	**Comment Category**	%*
What were the most positive aspects of the Brain Awareness visit?	Hands-on, age-appropriate	30
	Real human brain	23
	Knowledgeable/enthusiastic presenters	19
	Students were engaged/interested	16
	Good message	9
	Provided positive role models	3
Were there any negative aspects of the Brain Awareness visit? Please describe.	Presenter issues	42
	Need more time!	19
	Too much downtime	12
	Groups were too large	12

*Percentages are calculated based on the total number of comments for each question.

### Student open-ended responses

 Our first research question was addressed, in part, by analyzing the free response items from the 2010 survey. Almost 1600 student comments per open-ended question were analyzed and placed into nine categories; four categories specific to the activities themselves (touching brain, etc), three about the neuroscience content (parts of brains, neurons and how they work) and two about learning. As with the teachers, the students identified the opportunity to see and touch a real human brain and to learn about how the brain works as the most important parts of the Brain Awareness visits (Table 2). Forty-three percent of students cited content items as being the most important part of the visit, 35% cited the learning messages and 22% cited the activities themselves. In responding to the question of what they liked best, 88% of students listed various activities, with seeing and touching real brains being their favorite activities. 

**Table 2:** Student responses to open ended questions.

**Question**	**Comment Category**	**%**
What was the most important part of the brain visit?	Parts of the brain, how it works	32
	Learning about the brain	30
	Seeing the brain	12
	Protecting the brain	6
	General learning	5
	Neurons	5
	Other	4
	Puzzles and hands on activities	3
	Touching the brain	3
What part of the brain visit did you like best?	Touching a brain	34
	Seeing the brain	32
	Puzzles and hands on activities	18
	Parts of the brain, how it works	4
	Other	4
	Learning about the brain	4
	Neurons	2
	Protecting the brain	1
	General learning	1

The percentages are based on responses of ~1600 upper elementary school students from 2010.

### Student survey analysis

 Analyses of the 2011 student survey responses showed that presentations produced favorable shifts in student opinions on 16 of the 18 survey items. One item (#9) exhibited a negative trend and one had (#18) no pre- to post-survey change. Overall, these individual results are encouraging given the short, single presentation of the material 10 of the shifts represented statistically significant improvements, although the effect sizes were very small (*p*<0.05 or better, Cohen’s *d* range 0.06 - 0.13) (Table 3). Large effect sizes would not be expected in response to the brief nature of the classroom visits. Based on these responses to individual survey items, students responded more positively to items related to their enjoyment of science and knowledge of their own ability to grow intellectually. With respect to our most fundamental goal, the significant increases in agreement with statements 8 and 16, and decreased agreement with items 4 and 14 indicate that students understood and remembered the implications of the neuroscience content we presented.

**Table 3:** Individual item and factor analyses.

	Pre/post Comparison			Factor			
Survey item	*t*	*p*	*d*	1	2	3	4
10. Working as a scientist sounds fun to me.	2.07	.039	.06	**.754**	-.031	.027	.109
1. I enjoy science.	3.79	<.001	.11	**.710**	.035	-.012	-.108
13. I will probably take more science courses in school.	1.34	.182	.04	**.704**	-.067	.024	.094
11. I am good at science.	2.68	.007	.09	**.518**	.086	-.043	-.403
17. The science I have learned will help me in the future.	1.48	.139	.04	**.424**	.032	.222	-.021
5. I usually understand what we are doing in science class.	2.49	.013	.07	**.403**	.098	.042	-.284
4. You can learn new things, but you cannot really change how smart you are.	-2.57	.010	.07	-.10	**.632**	-.098	.122
14. I am already as smart as I can get.	-4.38	<.001	.13	.039	**.430**	-.140	.187
3. You either get science or you don't.	-.97	.330	.04	-.013	**.320**	.077	-.045
8. I think I can get smarter if I really try.	2.10	.036	.07	.034	.016	**.657**	-.004
16. With hard work, you can change how smart you are.	2.32	.02	.07	.044	.103	**.614**	.063
2. The harder I work at something, the better I’ll be at it.	1.35	.176	.04	-.005	.073	**.454**	-.071
7. I don't do well in science because I’m not a smart person.	-1.16	.246	.03	-.076	.103	-.017	**.573**
18. When I work hard at school, it makes me feel like I’m not very smart.	0.12	.901	0	.053	.073	-.105	**.454**
6. Scientists often do not have very good social skills.	-3.67	<.001	.11				
9. It is much more important for me to learn things in class than it is to get good grades.	-1.03	.305	.02				
12. The main thing I want when I do school work is to show how good I am at it.	-2.47	.013	.07				
15. Scientists usually work as part of a team.	0.52	.607	.02				

Individual item analysis (significance and effect sizes of pre-to-post survey differences) and the results of principal axis factoring with direct oblimin rotation for 2011. A four factor structure accounting for 56% of the variance resulted from the factor analysis. Values of *t* (two tailed t-test), *p* (associated significance level), and *d* (Cohen’s *d*, effect size) for each survey item. Factor loadings represent the degree of correlation between individual survey items and the clustered factors. Items loading onto each factor are shaded. Note the large absolute values among items that cluster into specific factors and the low absolute values for items not loading onto specific factors. For items in any one factor, note the low correlation values for the other factors, indicating a reasonably high degree of orthogonality. Items 6, 9, 12, and 15 did not load.

 Our survey was designed to gather data about multiple, underlying constructs, therefore, a factor analysis was employed to determine whether student responses were comparable on similar items. During the initial analysis, 4 of the 18 questions did not load highly enough in the pattern matrix, and are not included in the analyses presented below. Analysis of the remaining 14 items yielded a four-factor structure accounting for just over half (56.0%) of the variance. The factor loadings for the 2011 survey are listed for each statement in Table 3. Similar, but not identical, groupings were observed in the 2010 initial study (data not shown). Measures of internal reliability indicated good inter-item correlations among the items that loaded into factor 1 (Cronbach’s alpha = .81), and low but acceptable correlations for factors 3 (Cronbach’s alpha = .61) and 4 (Cronbach’s alpha =.57)[45]. Items grouped under factor 2 did not demonstrate significant correlation (Cronbach’s alpha = .47), therefore factor 2 items were excluded from further analyses. Neuroscientists (n=4) and education specialists (n=4) who were not associated with this project were asked to group the survey statements under these 4 factor headings. They placed 16 of the 18 items into the same categories as were indicated by the factor analyses (Factor 1: 5/6 statements were matched; Factor 2: 3/3, Factor 3: 3/3; Factor 4: 2/2; Items that did not load: 3/4).

Labels for the 3 factors that exhibited inter-item reliability were based on the statement or top two statements that exhibited the highest correlation(s) within each factor (Table 3):

Factor 1: Science is fun
Factor 3: I can get smarter
Factor 4: I don’t do well

**Figure 2:**
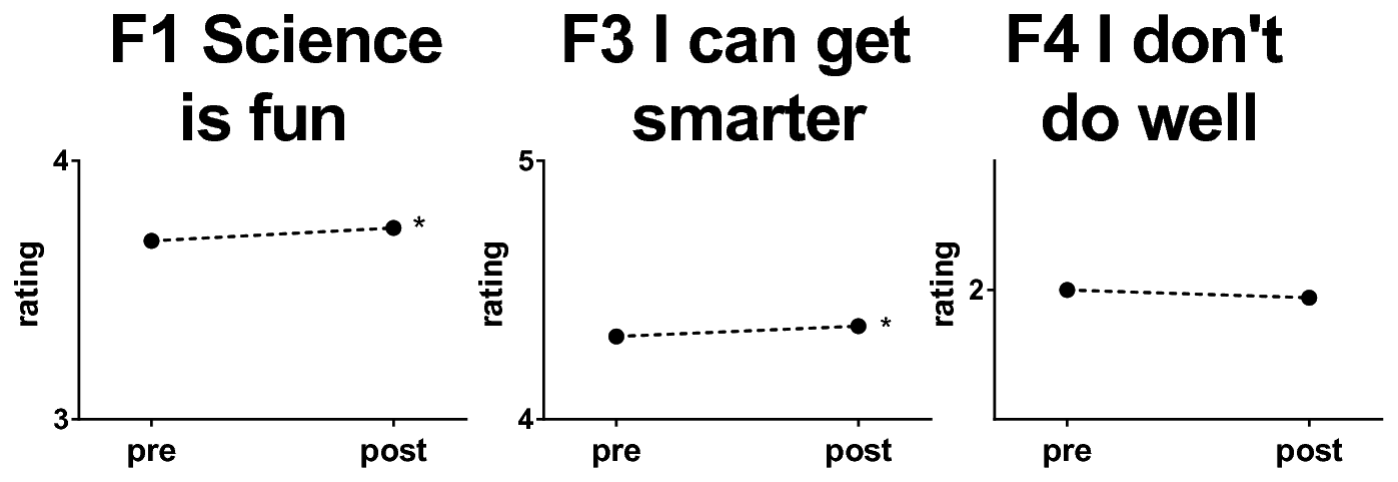
Changes in mean ratings on each factor from the 2011 surveys. Data are means from 4805 surveys. All y axes, on this and subsequent graphs, are one Likert unit high. Standard deviations range from 0.65 to 0.86 Likert units. * p<0.05, **p<0.01, ***p<0.001, 2 tailed independent samples *t*-tests. Cohen’s *d* for factors 1 and 3 were 0.07 and 0.24, respectively.

Comparisons between pre and post presentation mean responses were performed for factors 1, 3 and 4 by combining individual item responses (Figure 2). Following the Brain Awareness presentations, students showed significantly more agreement with statements related to the enjoyment of science (Factor 1) and their belief that hard work could cause their brains to change (Factor 3). The effect sizes (Cohen’s *d*) of the favorable changes in factors 1 and 3 were small, as was observed in the responses to individual items (Table 3). 

**Figure 3:**
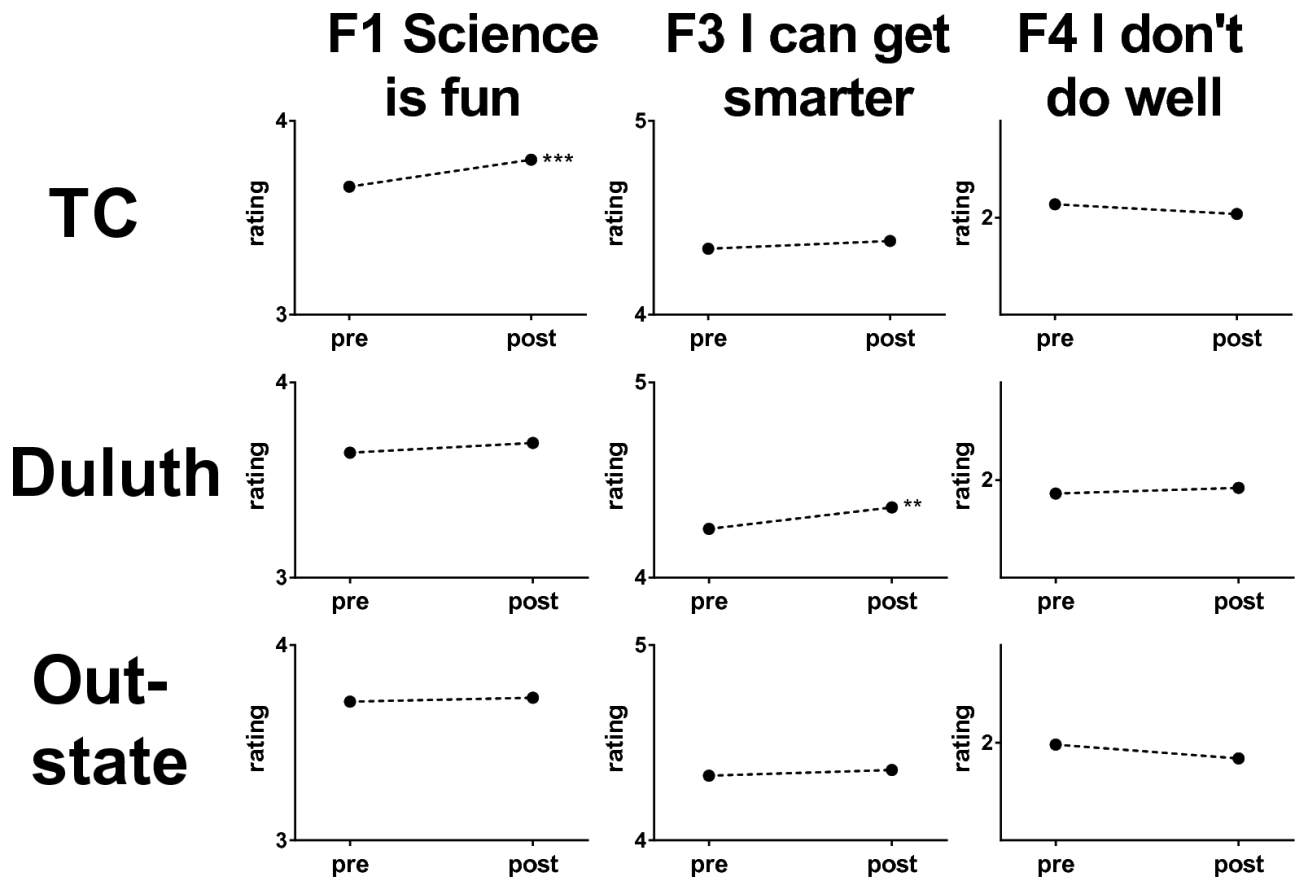
Changes in mean ratings on each factor from the 2011 surveys disaggregated by geographic region. N = 1819, 1184, 1802 responses from the TC, Duluth and outstate classrooms, respectively. Standard deviations range from 0.62 to 0.93 Likert units. Cohen’s *d* (effect sizes) for those factors with significant changes were: TC, factors 1 was 0.18; Duluth, factor 3 was 0.16. Y axes scales are one unit high. Significance as in Figure 1.

 We examined the data by geographic region to determine whether we were uniformly delivering our message. Analyses of the responses of students from three main geographic areas [the Twin Cities metropolitan region (TC), the city of Duluth and near environs (Duluth), and the cities and towns of greater Minnesota (Outstate)] were derived from the factor analysis described above (Figure 3). Differences in outcomes were evident, as the Twin Cities presentations resulted in significant changes in factor 1 (Science is fun) and in Duluth in factor 3 (I can get smarter). No significant changes in any of the 3 factors were observed in presentations made in outstate Minnesota. 

**Figure 4:**
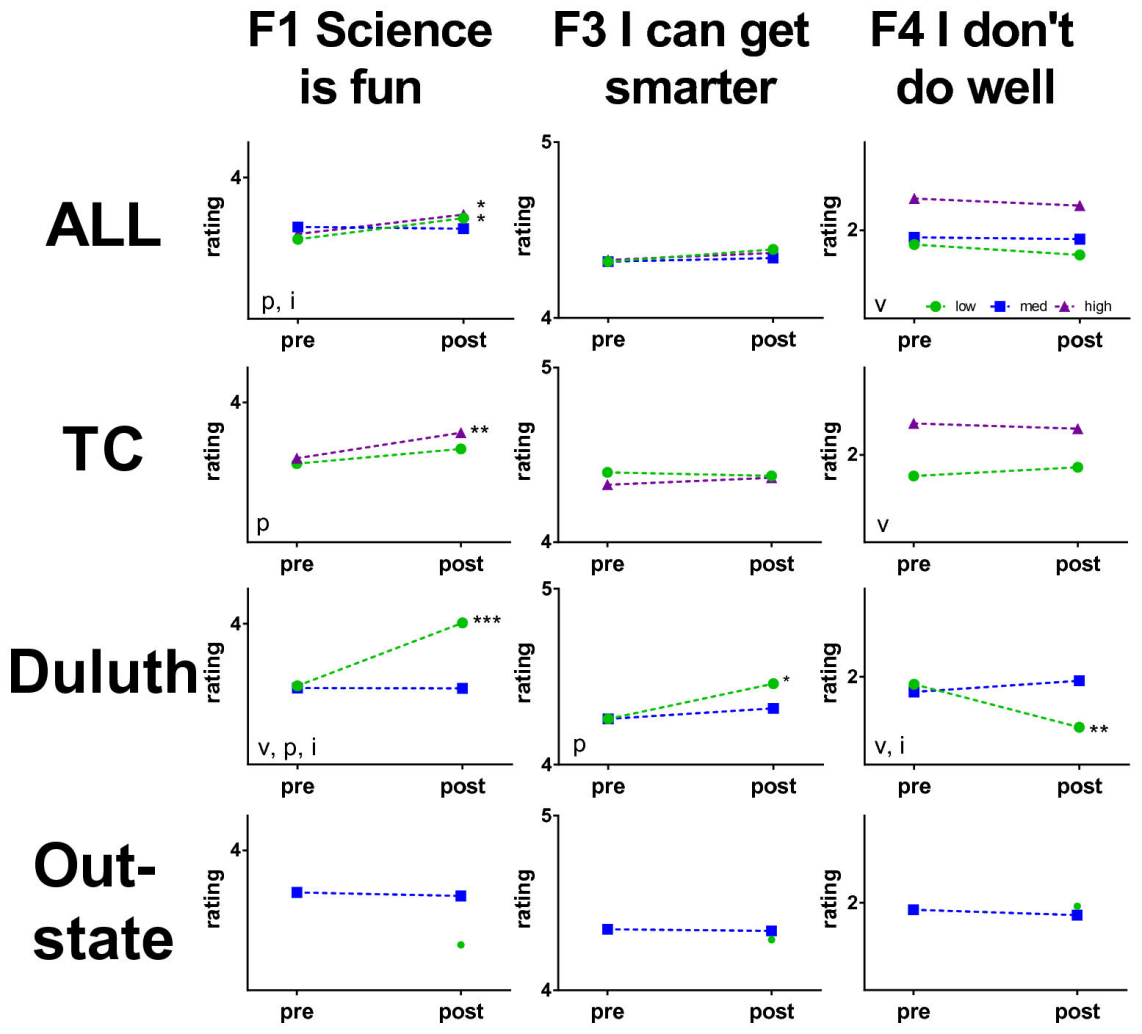
Changes in mean factor ratings disaggregated by school poverty and geographic region. All indicates entire aggregated 2011 data set N = 1311, 985, 1643 responses from the TC, Duluth and Greater Minnesota, respectively. V, p, and i indicate that significance in 2 by 3 (survey by poverty) ANOVAs within a region reached significance for poverty level, pre-post levels and their interaction, respectively at the p<0.05 or better level. *p<0.05, **p<0.01, ***p<0.001 indicate significance in post-hoc and simple effects tests with the Bonferonni correction. N’s as in Figure 1. Standard deviations range from 0.57 to 1.00 Likert units. Y axes scales are one unit high.

 A previous study indicated that students from schools in less affluent areas benefited more from a SiC visit than all students [37]. To determine if our BA presentations had a similar effect and to see whether differences in economic status mediated our regional differences in presentation effectiveness, we further disaggregated the survey data within each geographic region by the percentage of elementary students in each school receiving free or reduced price lunch (FRL) as a measure of social economic status. There was a substantial difference in poverty classification among surveyed schools in rural vs. urban regions of the state. Ninety-six percent of public schools visited in outstate Minnesota were characterized as medium poverty (0% high, 4% low), compared to a distribution of 29% low, 33% medium and 38% high poverty classifications among the urban public schools visited in TC and Duluth. Data were analyzed with two-way fixed effects ANOVAs followed by Bonferroni corrected simple effects tests (Figure 4).

 Among all classrooms visited, support for Factor 1 (Science is fun) significantly increased among students in low and high poverty schools following BA presentations (Figure 4, ALL). No significant changes in attitudes were detected for Factors 3 or 4 (I can get smarter, I don’t do well). In TC, BA presentations produced significantly more agreement with statements in Factor 1 in low poverty schools (Figure 4, TC). In Duluth, significant attitude changes for factors 1, 3 and 4 were observed in the more affluent schools (Figure 4, Duluth). Significant changes in responses among students in medium poverty schools were not observed across the state (Figure 4, Outstate and Duluth). Effect sizes for all of these comparisons were very small. Initially, students from high poverty schools agreed significantly more with the idea that “I don’t do well” than students in either low- or medium-poverty schools (Figure 4 Factor 4 ALL and TC). BA presentations had no impact on that belief in either the statewide or TC comparisons. The ability of students across the range of economic settings to respond to the BA presentations with changes in attitudes suggests that the message appeals broadly to all students. 

**Figure 5:**
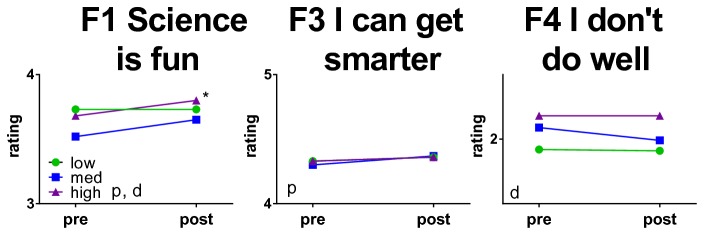
Changes in mean ratings on each factor from the 2011 surveys disaggregated by diversity rating of school. All indicates entire aggregated 2011 data set N = 3989 responses from the entire state. D, p, and i indicate that significance in 2 by 3 (survey by diversity) ANOVAs statewide reached significance for diversity level, pre-post levels and their interaction, respectively at the p<0.05 or better level. * p<0.05, **p<0.01, ***p<0.001 indicate significance in post-hoc Bonferonni adjusted tests. Standard deviations range from 0.64 to 0.91 Likert units. Y axes scales are one unit high.

 When the data were disaggregated by the percentage of minority students attending each school (Figure 5), regional differences were not disaggregated. Across the state, students in highly diverse schools significantly increased their agreement on Factor 1 and those in medium diverse schools trended in the same direction (Figure 5). The effect sizes of these comparisons were very small. As with the poverty analysis, schools in outstate Minnesota were considerably less diverse than urban schools, with 94% of participating schools being characterized as low diversity (1% medium and 0% high), compared to a distribution of 52% low, 17% medium and 31% high diversity among urban schools. 

## Discussion

 These data demonstrate clearly that a SiC program can have a positive impact on the learners’ by improving their enjoyment of science (factor 1), one dimension of attitudes towards science [32,33]. This was mirrored by teachers’ appreciation of the ability of the visits to stimulate student interest in science and their own brains. The well-received and remembered neuroscience content introduced students to nervous system concepts and impacted ideas about their own potential (factor 3). The lower level content messages (the wows and cool stuff that probably influenced factor 1) were more easily delivered than the higher level ones (“What does this mean for my own learning?” – ideas captured in factor 3). Ideas about doing poorly (factor 4) also diminshed among a subset of students. Among this group of students, movement in attitudes regarding their ability to apply themselves and grow accompanied reductions in self-identification as poor performers. Our assessment of student attitudes towards their own potential (factor 3) aligned with Dweck’s concept of a belief in incremental intelligence or a growth mindset [30,46]. The attitudes expressed in Factor 4 aligned with concepts of a negative self-efficacy. Students’ self-efficacy constitutes beliefs about their own abilities to perform well in school [47]. Dweck considers self-efficacy as a moderator, contributing to the attitude of a growth mindset, hence we incorporated these statements in this evaluation survey [46]. The exact relationship between concepts of self-efficacy and beliefs about intelligence, however, remain controversial [46,48]. Self-efficacy may mediate the impact of theories of intelligence on perceived performance [49]. How students view intelligence indirectly informs their beliefs about their self-efficacy and performance in science; views of malleable intelligence positively alter self efficacy, views of fixed intelligence negatively alter self efficacy [48]. Our study was not designed to investigate the distinctions and causal relationships between these concepts. Rather, in this evaluation of a single SiC visit, we found that both concepts could be altered, in line with previous, in depth investigations of the relationships between these ideas [46,48,49]. 

 These impacts may have depended on the characteristics of the audience. Agreement on statements indicative of positive views of science increased among students in more diverse environments and at both ends of the economic scale. For Factor 3, ‘I can get smarter’, students were very much in agreement with these concepts before the BA presentations. Registering positive change in this particular measure was more difficult since opinions were already on the upper range of the fixed scale. Similarly, most students initially disagreed with the negative statements in the ’I don’t do well’ Factor 4 category, leaving less room to further diminish agreement with these ideas. Movement on Factor 4 was only achieved among a subset of students in Duluth. Students in high poverty schools who had more room to move on this scale initially, were not impacted. 

 Student in schools classified as medium poverty did not register statistically significant changes in pre- vs. post-presentation comparisons. This group comprised a greater range (two quartiles) of free and reduced lunch recipients than either the high or low poverty groups (one quartile each). As such, some changes may not have been detected. Breaking this large category into separate quartiles may be needed to further analyze responses in greater Minnesota. Alternatively presenter training could have influenced this group (see below). Throughout the entire analysis, individual student poverty or ethnicity levels were not recorded. These breakdowns are by schoolwide reported statistics. So impacts can not be categorized based upon knowledge of individual circumstances or backgrounds. Overall, favorable changes in attitudes were obtained, both across the board, and among subpopulations of schools, demonstrating that one-hour classroom visits can impact student attitudes, at least in the short term.

### Variability and Training Differences

 Assessing a Scientist-in-the-Classroom program is a messy business. Everything about such programs is variable. Our program was no different. The presenters were not professional teachers and had little experience handling groups of energetic young learners. Presentations varied in the specific content covered and how well that content was delivered. The amount of time a presenter spent with a class varied by each school’s schedule. Some classrooms are visited annually, so teachers became familiar with the content and could prepare for the visit. Most of the classrooms hosted visits for the first time in 2010-2011, so teachers were unlikely to previously prepare students for the visit. The scientists had no idea if or how teachers followed up the visit. Students varied by age, ethnicity, demographics and background knowledge. Schools varied by their expectations, resources and administrative culture as well as their geographical and cultural context. Lesson success varied depending upon the time of day; attention spans differ before and after lunch or proximity to vacations or standardized testing. 

 Our Brain Awareness program embodied all of these variable elements and more. Our presenters varied in their degree of scientific background and education, from college undergraduates to senior scientists and in their mastery of presentation skills. Medical students also brought a different prospective on advanced education, focusing on their own goals to become doctors. In addition, specific training for classroom visits varied, depending upon the presenters’ campus affiliation. Materials left in the classroom varied depending upon choices made by staff in the two cities of origin. What every presenter had in common was an enthusiasm for communicating neuroscience to young audiences, a willingness to leave academic comfort zones to travel to schools and a desire to share information about brain function and health with lay audiences. 

 Since training records were not kept on each individual presenter, direct correlations between amount and type of training and classroom impact could not be computed. However, the geographical distribution of the regularly vs. irregularly trained presenters made it possible to extract some meaning from the data. The most gains on survey measures were registered among students in low poverty schools visited by the regularly trained, experienced presenters from Duluth. The Duluth presenters were retrained every year and their training emphasized the “synapses change” message. Only about a third of the presenters from the TC received training in the week before going to the schools as experienced TC presenters were not required to retrain. TC training was less about which activities to do and more about how to execute them to engage students in the activities. These differences predict that the message delivered by presenters trained in Duluth would be more uniform and on point. The data reveal that the synapses change message was internalized more in classrooms from presenters trained in Duluth, consistent with this dichotomy. TC presenters had a more consistent impact upon students’ enjoyment of science. The medical student presenters who visited outstate classrooms may have focused more on brain and mental health related issues, consistent with their own training, than on the content covered by survey items. The fidelity of the presentations constitutes a dimension where variability could be reduced. 

### Impact of SiC programs

 This study sets an example for scienctists engaged in public communication to begin to explore the impact of their efforts. Increased evaluation of public engagement efforts by scientists internationally has previously been recommended, but to date, mostly factors affecting scientists’ participation have been examined [50]. Not every educational intervention results in an increased attitudes towards science; undergraduate science majors can lose interest in prescribed material with little immediate application [51]. SiC programs typically are evaluated on the quality of the presentation and the skill of the presenters. Such formative evaluation is often not published or posted, although a few programs have provided online access as a measure of program success [52]. Information from our BA teacher survey, and similar ones like it from previous years (1998-2009), was used formatively to improve the activities, training sessions and content messages. This type of information provides sponsoring organizations with appropriate feedback to improve training, messaging and comportment of the staff that goes into schools, but does not evaluate the impact of presentations on the audience, as was done in this study. 

 SiC programs also have benefits for the presenters themselves. Interviews of graduate students in Science Squad, a biomedical outreach program with set curricula, determined that presenters felt they gained teaching and management skills that were transferable in future career placements, an understanding of educational issues and personal growth in self-confidence [8]. Previously, we polled medical student presenters for their assessment of the benefits of BA participation [53]. They liked sharing their enthusiasm for science, being ambassadors for the university, and found the experience contributed to their ability to explain basic disease concepts and body processes to future patients, and contribute to the community as a whole. “This experience…showed me a direct example of a way that I can, in my future small town practice, be a part of the community in a positive and useful way” [53]. Their experience is consistent with surveys of scientists, researchers and graduate students involved in science education outreach in Colorado, where the main motivation was a desire to contribute by sharing knowledge and to improve communication and teaching skills [54]. Additional motivating factors included a desire to dispel scientific misconceptions, to attract new scientists, and to have fun [54].

 Scientists engaged in public outreach are sometimes held in low regard by colleagues as these translational activities are considered of lower status than direct creation of new knowledge through research [55–57]. A careful analysis of the scientific output and public popularization activities of French researchers revealed just the opposite; the scientists most engaged in public dissemination were also those who were the most productive [57]. Of the hard scientists, those working in the subfields of behavior, cognition and brain were among the most active, with 59% engaged in 10 or more popularization activities annually [57]. Given that current data demonstrate a positive effect of SiC visits, the perceived professional credit for such outreach efforts should be elevated. 

Evaluations of the impacts of scientist classroom visits upon audiences are needed as scientists participating in such programs have previously indicated that “they would be more willing to participate in outreach if research showed that it was effective in increasing student knowledge and improving attitudes towards science” [54]. SiC visits are largely perceived by teachers, parents and students as providing students access to role models they would not necessarily encounter otherwise [8,37]. Analysis of programmatic impact in a large Scientists-in-Schools program in Canada reported a positive impact on ELL students, students from low achieving schools and girls in elementary schools despite the absence of overall impact across the entire population of schools visited [37]. This program resulted in ELL learners thinking more about careers in science and lower achieving students enjoying science more [37]. Female students and low achieving students reported that Scientists-in-Schools provided positive role models for them [37,38]. However, in a retrospective survey of the high school experiences of undergraduates, previous exposure to female scientists as guest speakers and role models was not a significant factor in establishing young women’s personal identity as a physicist and intended physics major [58].

More generally, evaluations have focused on long-term scientist-teacher partnership programs that build the stable relationships needed for systemic science education reform [41,59–61]. Teachers report that partnerships such as the former NSF GK-12 program, where graduate students acted as interns in K-12 classrooms, positively impacted learners knowledge of mathematics or science, interest in careers related to mathematics and science, analytical skills and knowledge of current findings from mathematics/science research [62,63]. None of these evaluations reported effect sizes. The small effect sizes reported in our study reflect the short length of the intervention. Larger effect sizes might be encountered from a longer intervention, considering how presenting real brains is a truly memorable experience.

### Public Understanding of Neuroscience

 The public is intrigued by the rapidly advancing field of neuroscience and what it tells us about ourselves [64]. While university-level teacher educators have been cautious about how much neuroscience knowledge should influence the educational process [65], teachers continue to search for ways to bring this content and the excitement of the research frontier to their students [66,67]. Direct contact between neuroscientists and young learners can build basic neuroscience literacy, increase interest in scientific endeavors and provide learners with a more positive understanding of their own potential. Progress demonstrated here in altering neuroscientific awareness among school aged children may eventually lead to an overall increase in public neuroscience literacy. The inclusion of neuroscience content in the new US Next Generation Science Standards will further this goal [68]. As the majority of UK teachers (and presumably those in the US and other countries as well) have no neuroscience in their backgrounds [69,70], scientist-in-the-classroom visits may initially play a large part in bringing this content into K-12 settings. True progress in achieving neuroscience literacy among all of these populations awaits larger scale, longitudinal surveys of public neuroscience knowledge [13,15,20]. 

### Study Limitations and Future Research

 The quasi-experimental pretest-posttest design was used because the natural classroom student groupings were not disrupted: individual children were not randomly assigned to groups. However, this type of design is more vulnerable to problems with internal validity because of the possibility of interactions between factors such as selection, maturation, history, and pretesting. In other words, posttest differences between groups may be attributable to characteristic differences between groups rather than to the intervention [71]. We addressed selection by assigning pre-presentation or post-presentation surveys randomly by classroom to the degree scheduling allowed. Maturation of subjects was not an issue in the short timeframe of this study. Individual student or class history was simply an unknown variable in our design. Pre-exposure to the survey did not contribute an effect as none of the post-presentation classrooms had previously seen the instrument. 

 This survey was partially successful in evaluating our initial goals. The open ended responses indicated that students did learn about brains, our primary goal. The factor analysis identified three clusters of survey items that appeared to represent distinct beliefs characteristic of interest in science, mindset and self-efficacy. These factors were internally consistent, had face validity and appeared to represent separate constructs. Although statements grouped under factor 4 were negative statements, these were not reciprocal concepts for the ideas in factors 1 and 3. A number of statements were discarded that did not align with the initial goals and did not load into the factor analysis. 

 Internal consistency is a necessary but not sufficient condition for determination of reliability and is only a small step on the long road to establishing construct validity [72]. From the analyses to date, however, the factors that arose out of our analyses speak to our original three research questions. We believe that these findings would benefit from additional psychometric investigation and revision in order to establish further reliability and validity. One way to better assess the construct validity of this instrument, for example, would be to include measures of concurrent and predictive validity [73], which would strengthen our argument that we significantly altered the mindset of elementary school children toward science. 

 The survey tried to cover too many ideas with not enough statements dedicated to each idea. For example, students’ responses to item 6, “Scientists often do not have very good social skills” showed a significantly decreased stereotype of scientists, yet it did not load in the factor analysis since the concept was distinct from those in other items. Hence a redesigned survey should add more statements focusing on fewer ideas. Another improvement might be to simplify the language, as some wording was perhaps too complex for the youngest students.

**Figure 6:**
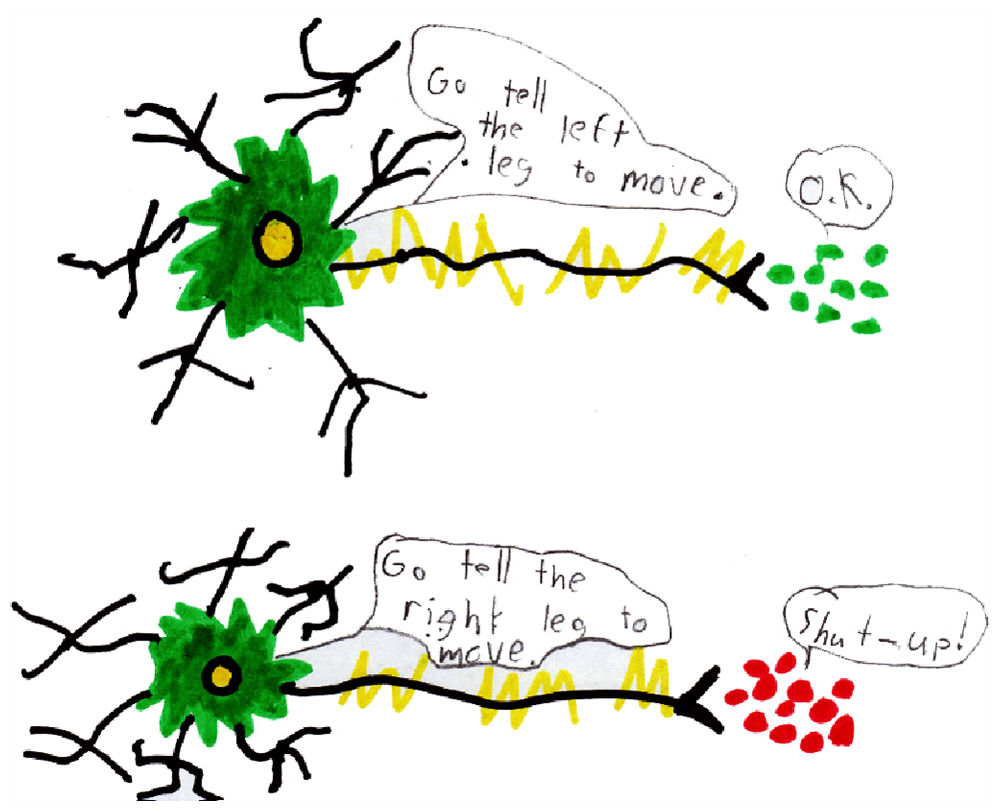
Sample of student work illustrating comprehension of the material in a BA visit. Concepts regarding neuronal structure, motor control and excitatory/inhibitor synapses, although presented separately, were combined into one drawing by the student.

 Small immediate attitude shifts occurred in response to BA SiC visits, especially among specific student populations. Whether these translate to long term impacts or true behavior changes remains unknown, but would likely require additional follow-up and emphasis from the classroom teacher. One benefit of administering the survey immediately after the visit, is that the responses reflect the impact of the visit unaltered by any additional classroom time spent on the brain as followup. The open-ended responses on the student surveys did not specifically test content learning but rather demonstrated student understanding and recall of the messages delivered during the BA visits. Content acquisition and longer term impacts, assessed at end of the school year and not immediately after the presentation, remain topics for future investigation. Anecdotal evidence was available in this study from thank you notes and drawings which often are received following a visit. One such drawing, received with a packet of surveys, graphically illustrates correct neuronal cytoarchitecture, electrical and chemical neuronal transmission, synaptic excitation and inhibition, nervous system control of movement and the concept of alternating movement of limbs, demonstrating that this student comprehended quite a bit of neuroscience (Figure 6).

 In view of the controversies surrounding the impact of neuroscience knowledge on the field of education, we want to emphasize that this study does not address why attitudes towards science should uniquely improve with more knowledge about the brain and learning. While we have no knowledge regarding whether SiC visits on other fun science topics would similarly positively impact student attitudes towards science, we strongly believe this would be expected from the other evaluations that have occurred to date [8,37]. Comparisons along these lines are now in order, since this study pioneered methodologies for quantitatively evaluating SiC presentations.

## Summary

Brain Awareness presentations had a positive effect on student attitudes toward science, boosting increased positive attitudes toward science and improved agreement with statements related to growth mindset. Impacts were greatest where presenters were experienced and well trained and in schools with more student diversity or high poverty. Overall effect sizes were small, consistent with the short length of the presentations. In conclusion, the impact of BA presentations was positive and proportional to the efforts expended, demonstrating that short, scientist-in-the-classroom visits can make a positive contribution to primary school students’ attitudes toward science and learning.
